# Editorial: New Approaches to the Pathogenesis of Sudden Intrauterine Unexplained Death and Sudden Infant Death Syndrome

**DOI:** 10.3389/fneur.2017.00441

**Published:** 2017-08-28

**Authors:** Anna M. Lavezzi, Conrad E. Johanson

**Affiliations:** ^1^“Lino Rossi” Research Center for the Study and Prevention of Unexpected Perinatal Death and SIDS, Department of Biomedical, Surgical and Dental Sciences, University of Milan, Milan, Italy; ^2^Department of Neurosurgery, Alpert Medical School, Brown University, Providence, RI, United States

**Keywords:** sudden infant death syndrome, fetal death, neuropathology, hypotheses, guidelines

**Editorial on the Research Topic**

**New Approaches to the Pathogenesis of Sudden Intrauterine Unexplained Death and Sudden Infant Death Syndrome**

Sudden Infant Death Syndrome (SIDS) is the leading cause of death among infants in the first year of age in the developed world. The better known definition of SIDS is the sudden unexpected death of an infant less than 1 year of age, with onset of the fatal episode apparently occurring during sleep, which remains unexplained after a thorough investigation, including performance of a complete autopsy and review of the circumstances of death and the clinical history (1). Despite the success of the “Back to Sleep” campaigns to reduce the risks encountered worldwide, the frequency of SIDS (striking one infant in every 750–1,000 live births) has not significantly declined in recent years (2).

Sudden Intrauterine Unexplained Death, referring to fetuses that die unexpectedly, particularly in the last weeks of gestation without any cause even after a complete autopsy, including examination of the placental disk, umbilical cord, and membranes, has a sixfold to eightfold greater incidence than that of SIDS (3). This death should be considered as a syndrome and referred to with the acronym “SIUDS,” “Sudden Intrauterine Unexplained Death Syndrome,” like “SIDS” (4). This definition is based on the observation that several conditions correlated with each other and occurring together may contribute to fetal death.

At this moment, the pathogenetic mechanism of these deaths has not yet been determined, even if the neuropathology seems to be a consistent substrate in both SIDS and SIUDS (5, 6). Subtle common developmental abnormalities of brainstem nuclei checking the vital functions have been highlighted, frequently related to environmental risk factors, such as cigarette smoke, air and water pollution, food contamination, etc. Exogenous toxic factors can also interact in complex ways with the infant genetic constitution, leading to polymorphisms and/or mutations of specific genes (such as polymorphisms of the serotonin transporter gene 5-HTT, the regulator of the synaptic serotonin concentration, and of the PHOX2B, the key gene in the Congenital Central Hypoventilation Syndrome) (7).

It is very important to stimulate research in this field to identify all the pathologic aspects and their correlation with exposure to environmental risk factors, in order to reduce the incidence of both SIUDS and SIDS, and mitigate the surrounding social concerns.

Therefore, we have launched this special Research Topic to collect innovative, still not yet considered approaches highlighting new possible pathological substrates, useful for planning specific prevention strategies to decrease the incidence of these unexpected and very devastating events for families and clinicians. Furthermore, the adoption of these preventive measures could also improve the quality of life in adults, promoting active and healthy aging.

In order to summarize the contributions, we have grouped the articles in two main sections.

(1) Original hypotheses
  New original interpretations of the pathogenetic mechanism leading to SIDS are proposed, assigning important causative roles to different issues. They are:
  (a) *SIDS–critical diaphragm failure (CDF) hypothesis*. The basic premise of this hypothesis is that the diaphragm is a vital organ that must continuously generate adequate force to maintain ventilation. Consequently, a CDF, caused by different combinations of factors, can be the terminal event leading to SIDS (Siren).
  (b) *Microbiome–Gut–Brain axis hypothesis*. This hypothesis is based on the consideration that the infant’s gut microbiome, with its metabolites, is able to stimulate the afferent gut vagal endings and consequently to modulate the brainstem 5-HT level (the so-called “microbiome–gut–brain axis”). A decreased release of serotonin, concomitant with critical periods of gut flora development and infant vulnerability, could play a significant role in SIDS (Praveen and Praveen).
  (c) *“Wear and Tear” hypothesis*. This hypothesis proposes that SIDS is the result of cumulative painful, stressful, or traumatic exposures that begin *in utero* and, after birth, lead to allostatic overload with consequent impairment of the vital regulatory systems (Elhaik).
  (d) *Acute respiratory infection and anemia hypothesis*. Through the use of probability models, an association between an occult prodromal respiratory infection and unmeasured asymptomatic anemia, is proposed as a possible cause of SIDS. The maternal iron-deficiency anemia *in utero*, in particular, plays a causative role in severe physiological anemia associated to delayed neurological development *ex utero* (Mage et al.).
  (e) *Fetal reflex awake hypothesis*. During the first months of life, in hypoxic conditions, a particularly vulnerable infant can unexpectedly awaken an ancestral fetal behavior aimed to suspend respiration to save energy, as happens *in utero*, when breathing is not an essential activity. This mechanism represents a protective reflex in the womb but rapidly leads to a fatal outcome in postnatal life (Lavezzi).
(2) Guidelines
  (a) Indispensable procedures for the examination of the young victims are recommended, above an in-depth histopathological analysis of the cardiac conduction system and autonomic nervous system performed by specialized pathologists (Alfonsi and Crippa; Ottaviani).
  (b) The neuropathological study must be focused particularly to specific nuclei and structures of the brainstem checking the vital functions and whose development is frequently compromised in SIUDS and SIDS (Mehboob et al.).
  (c) Care, in the context of the neuropathological examination, should be given even to the cerebellar cortex, with particular attention to Purkinje cells. Developmental loss of these cells, in fact, seems to be involved in reduced compensatory response to the increased body burden of CO_2_ (Calton et al.).
  (d) Application of immunohistochemical techniques is advocated to highlight the specific involvement of the catecholamine and trigeminal systems in SIDS. Alterations of the immunoreactivity for tyrosine hydroxylase in the dorsal vagal nucleus and ventrolateral reticular formation in the medulla oblongata, and for the neuromodulator Substance P and its receptor Neurokinin 1 in the spinal trigeminal nucleus, may lead to disturbed autonomic regulation and/or respiratory control, so providing a possible explanation of SIDS (Hayashi and Sakuma; Mehboob).
  (e) Among the *risk factors* for sudden perinatal deaths, worthy of note is the proposal to systematically search, by means of toxicological analysis of brain samples concomitant with the autopsy, the endocrine disruptors (EDs), a wide group of organic chemicals able to persist in the environment for a long time due to their degradation resistance (Roncati et al.). EDs are able to interfere with the endocrine system, so affecting important biological processes, especially when exposure occurs during early life stages (8).

Our hope, in conclusion, is that all these contributions can be useful to expand the current knowledge on SIUDS and SIDS, thus allowing the broadening of the diagnostic criteria and preventive strategies.

As editors of this Research Topic, we would like to express our sincere gratitude to all of the authors who accepted the invitation to participate and for their significant efforts for identifying very interesting approaches to explain the pathogenesis of these pathologies.

We would like also to thank the reviewers, for their significant comments, useful for the improvement of the submitted articles. Finally, we thank Frontiers and, in particular, its Editorial Office for their competent and essential support.

## Author Contributions

AL and CJ are the editors of the research topic “New approaches to the pathogenesis of sudden intrauterine unexplained death and sudden infant death syndrome.” Together they wrote the editorial that summarizes all the contents of the twelve contributions.

## Conflict of Interest Statement

The authors declare that the research was conducted in the absence of any commercial or financial relationships that could be construed as a potential conflict of interest. The reviewers, KS and SC, and handling editor declared their shared affiliation.
